# Posterior Hip Dislocation Associated with Posterior Wall Acetabular Fracture and Ipsilateral Intertrochantric Fracture: a Very Rare Case Report

**DOI:** 10.5812/traumamon.8520

**Published:** 2013-01-15

**Authors:** Ahmad Yousefi, Hami Ashraf, Ali Mashhadinezhad, Ali Birjandinejad

**Affiliations:** 1 Orthopedic and Trauma Research Center, Shahid Kamyab Hospital, Mashhad University of Medical Sciences, Mashhad, IR Iran

**Keywords:** Hip Dislocation, Fracture, Posterior, Sciatic Nerve

## Abstract

**Abstract:**

Traumatic hip dislocations are common in high-energy motor vehicle accidents. We present a case of a 43-year old man who sustained posterior hip dislocation with posterior wall acetabular fracture and ipsilateral intertrochantric fracture following a motorcycle accident. Urgent open reduction and internal fixation of the hip fracture-dislocation and fixation of intertrochantric fracture with a dynamic hip screw were done. To our knowledge, such an injury has been rarely reported in the literature. Possible mechanisms of injury and operative procedures are discussed. Radiographic follow-up after eight months showed union. No major complications were observed in our patient.

## 1. Introduction

The hip joint is one of the most stable joints in the body and its dislocations usually occur following high-energy road traffic accidents. Because massive force is required to dislocate the hip, screening for associated injures should be considered in these dislocations. Femoral head or acetabulum fractures are commonly associated fractures in hip dislocation. We describe a case of posterior hip dislocation with posterior wall acetabular fracture and ipsilateral intertrochantric fracture following a motorcycle accident. Not many cases similar to ours was found in the English literature.

## 2. Case Report

**Figure 1:**
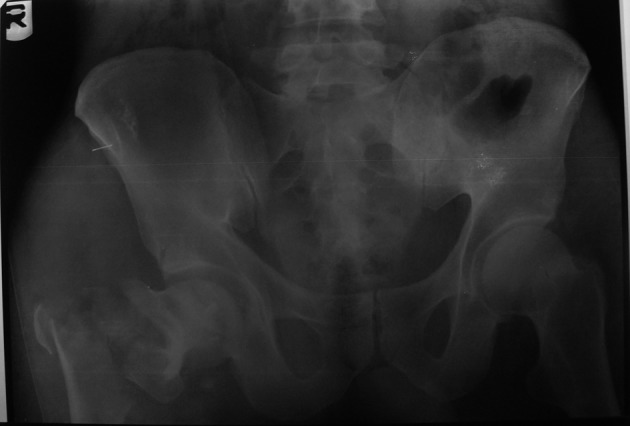
Radiograph Taken on Admission

**Figure 2:**
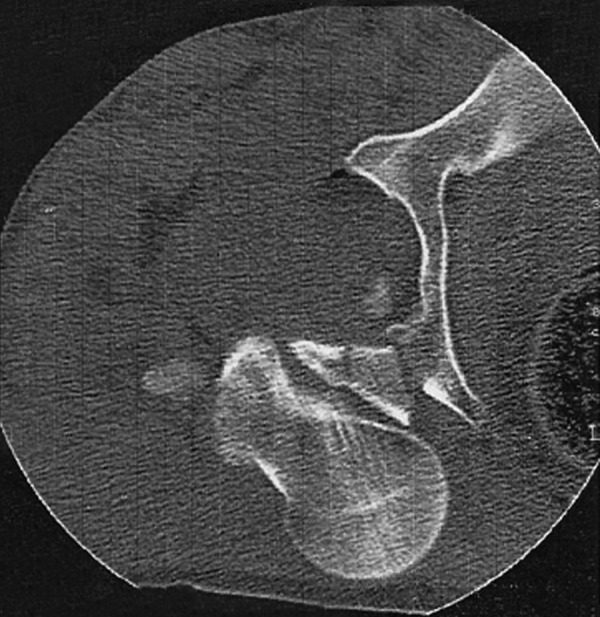
Computed Tomogram on Admission

In May 2010, a 43-year-old farmer who was riding a motorcycle, collided front-to-side with a pickup and was thrown from his bike. He was admitted 2 hours later to our hospital. Upon admission, he was conscious (GCS = 14) and hemodynamically stable. He had severe pain in the right hip and knee and was unable to move his right leg. On examination, his right leg was 2 cm shortened and rotated externally without any open wound. Swelling with abrasions on the right knee and ecchymosis around the left eye and on the posterior of left shoulder were observed. Vascular examination was normal. Upon neurological exam, he was unable to dorsiflex his toes in the right foot and sensory touch was impaired in the dorsal and plantar regions. Examination of the trunk and other limbs showed no injury. Plain radiographs of the pelvis (Figure 1) revealed a posterior hip dislocation with posterior wall acetabular fracture and ipsilateral intertrochantric fracture. There was no fracture of the femoral head or neck. Brain CT scan and radiographs of the knee and shoulder were normal. Computed tomography (CT) scan of the hip confirmed the findings (Figure 2).

**Figure 3:**
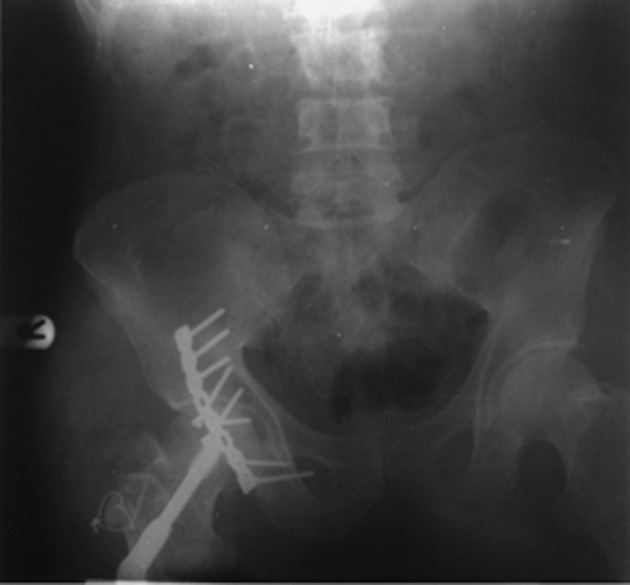
Radiograph After Eight Months

Considering dislocation and fracture type, no attempt was made for closed reduction. Urgent surgical reconstruction of the fracture dislocation was performed four hours after injury under general anesthesia in a lateral position via a posterolateral approach. Intraoperatively, the sciatic nerve was intact but compressed by a fragment of bone and the hip capsule was ruptured posteriorly. The comminuted acetabular fracture was reduced and fixed with a reconstructive plate and cortical screws. Then on the orthopedic table in supine position with extension of the incision to lateral of the femur, the intertrochantric fracture was reduced and fixed with dynamic hip screw using C-arm imaging. After ten days the patient was discharged and after three weeks partial weight bearing walking was permited with crutches. At last follow-up after eight months, no radiological sign of avascular necrosis was seen (Figure 3). The movements at the hip were terminally restricted and dorsal right foot remained numb.

## 3. Discussion

Hip joint injuries during traffic accidents can range from mild trauma without a major functional problem to more severe types of fractures and fracture-dislocations of the hip. Posterior dislocations account for approximately 90% of hip dislocations (1). Position of the hip at the moment of impact and vectors and intensity of the forces affect the direction of the dislocation and whether a fracture-dislocation or a pure dislocation occurs (2). During a motor vehicle accident, if the axial forces are applied through the femur while the hip is flexed and adducted, posterior hip dislocation usually occurs. Slight degrees of hip adduction at the time of collision, also leads to posterior wall fracture of the acetabulum. Further adduction of the femur head fixed by the tight periosteum of the ilium can cause the fracture of the femoral neck (3). In our patient, we believe that the mechanism was axial force during the collision while the hip was in adduction and caused hip fracture-dislocation. This was followed by an external rotation force which led to an intertrochantric fracture. Another possible mechanism could be a hip fracture-dislocation caused by a longitudinal compressive force combined with adduction followed by direct force in the throcantric region when thrown off the bike. Such a fracture pattern has not been explained by the classification systems described for hip dislocation injuries (4, 5).

In reviewing English literature, associations between hip dislocations and ipsilateral trochanteric fractures are extremely rare revealing only four case reports (6-9). Although the first approach consisted in an attempt for close reduction in some case reports, most authors recommended an open reduction. Considering the emergency of such injuries, in addition to the severity of damage, duration of the dislocation has a considerable impact on patient outcome. Reduction within six hours after trauma showed better results (10). The incidence of nerve injury in hip dislocations is approximately 10% in adults (11). The sciatic nerve, especially the peroneal division, is a common nerve injury in traumatic posterior hip dislocations associated with displaced posterior acetabular fractures. Despite varying reports of nerve injury outcomes in traumatic hip fracture-dislocations, return of nerve function can be expected in approximately 60% to 70% of patients (11). Some authors (12-14)recommend immediate open reduction and surgical exploration of the sciatic nerve in such cases. Others (4, 15) believe in attempting a closed reduction of displaced fracture-dislocations with conservative treatment of the nerve injury after a successful reduction of the all acetabular fragments. Considering neurologic deficit in our patient with such an unusual fracture pattern, urgent open reduction was done to prevent further possible injury. At last follow-up eight months later, dorsal numbness of the right foot still remained so we considered conservative treatment. Plain radiographs of the pelvis showed union and normal anatomy without signs of avascular necrosis of the femoral head. This can be somewhat related to reduction within the “golden time” period. Although longer follow-up is needed, we can rule out the possibility of avascular necrosis and other related complications.

## References

[1] Lamberti PM, Rabin SI (2003). Open anterior-inferior hip dislocation.. J Orthop Trauma..

[2] Hougaard K, Thomsen PB (1988). Traumatic posterior fracture-dislocation of the hip with fracture of the femoral head or neck, or both.. J Bone Joint Surg Am..

[3] Fernandes A (1981). Traumatic posterior dislocation of hip joint with a fracture of the head and neck of the femur on the same side: a case report.. Injury..

[4] Stewart MJ, Milford LW (1954). Fracture-dislocation of the hip; an end-result study.. J Bone Joint Surg Am..

[5] Thompson VP, Epstein HC (1951). Traumatic dislocation of the hip a survey of two hundred and four cases covering a period of twenty-one years.. The Journal of Bone and Joint Surgery (American)..

[6] Agarwal R, Rai AK, Saraf SK, Singh S, Singh A (2008). An unusual pattern of posterior dislocation of hip associated with comminuted trochanteric fracture.. The Internet Journal of Orthopedic Surgery..

[7] Barquet A, Mussio A (1983). Fracture-dislocation of the femoral head with associated ipsilateral trochanteric and shaft fracture of the femur.. Arch Orthop Trauma Surg..

[8] Singh R, Sharma SC, Goel T (2006). Traumatic inferior hip dislocation in an adult with ipsilateral trochanteric fracture.. J Orthop Trauma..

[9] Alexa O, Puha B, Veliceasa B, Păduraru D (2009). Posterior dislocation of the hip associated with ipsilateral trochanteric fracture--a very rare case.. Chirurgia (Bucharest, Romania: 1990)..

[10] Durakbasa O, Okan N, Canbora K, Gorgec M (2005). [Factors affecting the results of treatment in traumatic dislocation of the hip].. Acta Orthop Traumatol Turc..

[11] Cornwall R, Radomisli TE (2000). Nerve injury in traumatic dislocation of the hip.. Clin Orthop Relat Res..

[12] Armstrong JR (1948). Traumatic dislocation of the hip joint: Review of one hundred and one dislocations.. JBone Joint Surg..

[13] Bromberg E, Weiss AB (1977). Posterior fracture dislocation of the hip.. South Med J..

[14] Wilson JN (1960). The management of fracture dislocation of the hip.. Proc R Soc Med..

[15] Proctor H (1973). Dislocations of the hip joint (excluding ‘central’dislocations) and their complications.. Injury..

